# β2GPI-targeted polymeric nanoparticles form a protective layer to prevent vascular thrombosis in an anti-phospholipid syndrome model

**DOI:** 10.3389/fimmu.2025.1520619

**Published:** 2025-02-27

**Authors:** Paolo Durigutto, Maria Cristina Grimaldi, Sara Bozzer, Elena Raschi, Pierluigi Meroni, Francesco Tedesco, Paolo Macor

**Affiliations:** ^1^ Laboratory of Immuno-Rheumatology Istituto Auxologico Italiano, Istituto di Ricovero e Cura a Carattere Scientifico (IRCCS), Milan, Italy; ^2^ Department of Life Sciences, University of Trieste, Trieste, Italy

**Keywords:** nanoparticles, thrombosis, anti-phospholipid syndrome, β2GPI, rat model

## Abstract

Anti-phospholipid syndrome (APS) is a systemic autoimmune disease characterized by thrombotic vascular occlusion and maternal morbidity. Anti-coagulants remain pivotal drugs for the management of APS, but a significant proportion of patients do not benefit from long-term anti-coagulation and may require an alternative therapy to prevent antibody deposition and vascular thrombosis. We have developed a therapeutic approach based on the use of safe polymeric nanoparticles that selectively target beta2-glycoprotein I (β2GPI) deposited on endothelial cells (tNPs). Their efficacy was tested in a rat model of APS developed by infusing patients’ sera containing medium–high titer antibodies against domain I of β2GPI. The tNPs bearing a CH2-deleted anti-β2GPI recombinant antibody as a targeting agent recognize β2GPI deposited on endothelial cells but failed to induce blood clot formation. The tNPs infused into rats immediately before APS sera competed with patients’ antibodies, preventing their binding to deposited β2GPI and, as a consequence, resulted in thrombus formations and occlusion of mesenteric vessels. Similar results were obtained by injecting tNPs 24 hours before the administration of patients’ sera to induce blood clot formation. Our findings suggest that β2GPI-targeted polymeric nanoparticles represent a stable and safe approach to prevent thrombus formation and vessel occlusion in a rat model of APS and may be used to control thrombosis developing in APS patients as a result of acute triggering events.

## Introduction

Vascular thrombosis and pregnancy complications are the main clinical manifestations of anti-phospholipid syndrome (APS), an autoimmune disease that affects relatively young individuals with important clinical and socio-economic implications (1). Although blood clots can form in arteries and veins of different sizes in the vascular tree, they tend to develop more often in vessels of some districts, leading to stroke, deep vein thrombosis, pulmonary embolism, and, less frequently, myocardial infarction (2). Occasionally, the disease manifests as catastrophic APS (CAPS), a very aggressive variant of the syndrome, characterized by small-vessel occlusion and multiple organ failure that develops over a short period of time (3, 4).

Anti-phospholipid antibodies (aPL) detected in the sera of patients with APS are mainly directed against beta2-glycoprotein I (β2GPI), currently considered the most relevant target antigen of aPL (5, 6). The anti-β2GPI antibodies, now included among diagnostic tests and classification criteria of the disease, have been shown to play a key role in the development of vascular thrombosis and pregnancy loss. This conclusion is supported by the finding that IgG, purified from the sera of patients with medium–high titers of anti-β2GPI antibodies, were able to induce thrombus formation in a rat model of APS and that the prothrombotic effect was prevented by the depletion of anti-β2GPI antibodies via affinity chromatography (7). The management of patients with APS, aimed at preventing vascular thrombi, is based on the use of anti-coagulant drugs, such as heparin or warfarin, and a combination of low-dose aspirin and low-molecular-weight heparin (8, 9). However, despite the satisfactory results obtained with these preventive measures, a significant proportion of patients do not benefit from long-term anti-coagulant therapy and develop recurrent thrombosis (1, 10). Drugs that interfere with the immune mechanism responsible for thrombus formation in APS, starting from the binding of antibodies to their target antigen β2GPI and proceeding with complement activation, represent an alternative therapeutic strategy. Complement inhibition with eculizumab (Soliris) has been recommended for the treatment of patients with CAPS, following anecdotal reports of beneficial effects of this antibody in a few patients refractory to standard treatment (11, 12). However, there is no general consensus on the use of complement inhibitors as first-line therapy in these patients (13). Inhibition of antibody binding to β2GPI is another therapeutic option that can be obtained with a monoclonal antibody that competes with the patients’ antibodies, preventing their deposition on cell-bound β2GPI. We have previously shown that this approach is feasible using an anti-β2GPI single-chain fragment variable isolated from a human phase display library and subsequently engineered to contain an IgG1 hinge-CH2-CH3 domain (MBB2) (14). This antibody recognizes the DI domain of β2GPI with a higher affinity than patients’ antibodies, and the CH2-deleted variant of this antibody (MBB2ΔCH2) was shown to prevent their ability to induce thrombus formation in a rat model of APS (13).

We now present a therapeutic approach to prevent thrombotic vascular occlusion using nanoparticles coated with MBB2ΔCH2 to target β2GPI deposited on the endothelium, allowing the β2GPI-targeted nanostructures to act as a protective shield against the prothrombotic effect of patients’ antibodies in a rat model of APS.

## Materials and methods

### Nanoparticle preparation and characterization

Poly(lactic-co-glycolic) acid (PLGA)–polyvinyl alcohol (PVA) nanoparticles (NPs) were synthesized by the double-emulsion solvent evaporation method described by Vasir and Labhasetwar (15). Briefly, PVA (1 mg/mL in 2-Morpholinoethanesulfonic acid monohydrate (MES) buffer) (Sigma-Aldrich Co., St. Louis, MO, USA; m.w. 30,000–70,000, 87%–90% hydrolyzed) was covalently linked to MBB2ΔCH2 (300 μg) through the EDC/sulfo-NHS Crosslinking protocol (Thermo-Fisher Scientific, Waltham, MA, USA) and then added to 10 mL of 2.0% (w/v) PVA solution in cold Tris-EDTA pH 8 (TE buffer). Afterward, 30 mg of PLGA (Sigma-Aldrich Co.; m.w. 30,000–60,000, lactide:glycolide 50:50) were dissolved in 1 mL of chloroform. To start the synthesis, 300 μL of bovine serum albumin (BSA) or Fluorescein isothiocyanate (Fitc)-BSA (Sigma-Aldrich Co.; 20 mg/mL) were added to the PLGA solution and vortexed and sonicated for 30 s at 15 W of the maximum power using Bandelin Sonopuls HD 2070 (Bandelin electronic GmbH & Co. KG, Berlin, Germany) to form the primary emulsion. The emulsion was added to the PVA solution and sonicated for 1 minute as described above to form a secondary emulsion. The resulting emulsion was stirred (750 rpm) overnight to allow for chloroform evaporation. NPs were collected by centrifugation at 12,000*g* for 30 minutes at 4°C, and the pellet was washed in TE buffer. Finally, NPs were resuspended in 2 mL of phosphate-buffered saline (PBS) filtered 0.2 μm and stored at 4°C. Untargeted NPs were produced following the same protocol, skipping the antibody coupling step.

### Nanoparticle tracking analysis

Nanoparticle tracking analysis (NTA) was performed using NanoSight LM10 (Malvern Panalytical, Malvern, UK) diluting NP samples 1:4,000 (v/v) in Milli-Q water immediately before the measurements.

### Dynamic light scattering

Hydrodynamic diameter (Z-Ave), polydispersity index (PDI), and surface charge (zeta potential) analyses were performed with the dynamic light scattering technique using Zetasizer Nano ZSP (Malvern Panalytical, Malvern, UK). NPs were diluted 1:200 (v/v) in H_2_O Milli-Q filtered 0.2 μm, and measurements were performed at 37°C and a scattering angle of 173°.

### Scanning electron microscopy

The morphology of NPs was analyzed by scanning electron microscopy (SEM). NPs were diluted 1:50 v/v in H_2_O Milli-Q filtered 0.2 μm. Then, a drop of the sample was deposited on a glass coverslip and left to dry at room temperature. Glass coverslips were mounted on aluminum stubs coated with double-sided carbon tape. Samples were carbon coated using the Q150T ES plus sputter coater (Quorum, Sacramento, CA, USA). Samples were analyzed by scanning electron microscopy using a Gemini 300 SEM (Zeiss, Oberkochen, Germany), working in secondary electron mode, at an acceleration voltage of 2 kV and a working distance of 3 mm.

### CH50 screening assay

Functional activity of complement was determined by measuring the activity of the classical pathway (CH50) on sensitized mutton erythrocytes (EA, Microbiol s.r.l., Cagliari, CA, Italy) as previously described (16, 17). Briefly, NHS (100 μL) and NPs (8 μL) were incubated for 2 hours at 37°C under shaking (750 rpm). The samples were centrifuged for 5 minutes at 8,000*g*, and the supernatants were diluted in complement fixation diluent (CFD; NaCl 142 mM, Na-5-5-diethylbarbiturate 5 mM, MgCl_2_ 0.5 mM, agar 0.05%, NaN_3_ 0.01%, and Ethyleneglycol- bis(β-aminoethyl)-N,N,Nʹ,Nʹ-tetraacetic Acid (EGTA) 10 mM) at 1:50, 1:100, 1:200, and 1:400 (v/v) in a final volume of 200 μL and incubated with 50 μL of antibody-sensitized erythrocytes (EA) 1% for 30 minutes to activate the classical complement pathway. The total lysis is represented by EA 1% diluted in H_2_O, while the blank is represented by EA 1% diluted in CFD. The reaction was then blocked by the addition of EDTA 20 mM. The % lysis was measured at 415 nm with ELISA Reader TECAN Infinite M200 (Tecan Italia, Milan, Italy) after centrifugation at 12,000*g* for 1 minute. Complement activation was checked by preparing a standard curve using NHS as a positive control. The % lysis was calculated using the following formula:


$$\%\text{Lysis}\;=\frac{\text{O}{\text{D}}_{415}\text{Sample}-\text{O}{\text{D}}_{415}\;\text{Blank}}{\text{O}{\text{D}}_{415}\;\text{Total}\;\text{Lysis}\;-\;\text{O}{\text{D}}_{415}\;\text{Blank}}*100.$$

The % lysis versus the serum dilution was plotted, and the dilution required for 50% hemolysis was calculated. The CH50 (U/mL) (hemolytic complement 50) provides the number of hemolytic units present in mL of NHS.

### Hemolytic assay

Direct erythrocyte cell lysis was performed by incubating EA 1% with NPs (8 μL) diluted in CFD for 2 hours at 37°C under shaking (750 rpm). The % lysis was calculated as described in the section “CH50 screening assay”.

### Clotting test

NPs (4 μL/well) and normal human plasma (NHP; 80 μL/well) were seeded in a 96-well plate. To assess the clotting capacity of the nano-devices, CaCl_2_ 20 mM was added to initiate the clotting reaction. The eventual coagulation causes an increase in the turbidity of the well, which has been read at 405 nm every 2 minutes for 70 minutes with ELISA Reader TECAN Infinite M200 (Tecan Italia S.r.l.).

### Patients’ sera and recombinant antibody to β2GPI

Serum samples were obtained from five APS patients (**Table 1**) with medium–high titer antibodies to domain I of β2GPI after obtaining informed consent and were previously shown to induce clot formation in rats (18). Normal human serum was the pool of at least 30 different healthy donors. NHP was the pool of at least 30 different healthy donors using EDTA as an anti-coagulant. The ethical committee of Istituto Auxologico Italiano approved the study; the patients/participants provided written informed consent to participate in this study. Total IgG were purified from human sera using protein A affinity chromatography and then labeled with Fitc, as already described by our group (19).

**Table 1:** Characterization of APS sera.

ID	Diagnosis	CAPS	aCL IgG	aCL IgM	aβ2GPI IgG	aβ2GPI IgM	aD1 IgG	LAC
1	PAPS	No	**Medium Pos**	Neg	**Medium Pos**	Neg	**Pos**	Neg
2	PAPS	No	**High Pos**	Medium Pos	**High Pos**	Medium Pos	**Pos**	Pos
3	SLE-APS	No	**High Pos**	high hos	**High Pos**	high Pos	**Pos**	Pos
4	PAPS	Yes	**High Pos**	Medium Pos	**High Pos**	Medium Pos	**Pos**	Pos
5	SLE-APS	No	**Medium Pos**	Medium Pos	**Medium Pos**	Medium Pos	**Pos**	Neg

Clinical status of the patient source of the sera used in the *in vivo* experiments and *in vitro* characterization of the antiphospholipid reactivity of the sera.

PAPS, primary anti-phospholipid syndrome; SLE, systemic lupus erythematosus; CAPS, catastrophic anti-phospholipid syndrome; aCL, anti-cardiolipin IgG or IgM; aβ2GPI, anti-beta2 GPI IgG or IgM; aD1, anti-domain I of beta2 GPI IgG; LAC, lupus anticoagulant; Pos, Positive.Bold values indicate IgG results that are more important than IgM for our study.

MBB2ΔCH2 was produced as previously described (14). Briefly, stable transfected CHO cells were cultured in a serum-free medium, and the recombinant antibody was purified from cell supernatant by protein A affinity chromatography.

### Animal models

*In vivo* experimental models were established in groups of three male Wistar rats (290–320 g) kept under standard conditions in the Animal House of the University of Trieste, Italy. The *in vivo* procedures were performed in compliance with the guidelines of European (86/609/EEC) and Italian (Legislative Decree 116/92) laws and were approved by the Italian Ministry of University and Research (Prot. No. 910/2018PR). The study was conducted in accordance with the Declaration of Helsinki.

### Binding of tNPs, IgG from APS patients, and MBB2ΔCH2 to β2GPI on rat vascular endothelium

Rats were intraperitoneally injected with lipopolysaccharide (LPS) (2.5 mg/kg) and then anesthetized with Zoletil (Virbac, Carros, France; 25 mg/kg) and xylazine (Rompun; 7.5 mg/kg). Afterward, they received a slow infusion of Rhodamine 6G (Sigma) into the jugular vein to stain leukocytes and platelets. Targeted or untargeted BSA-Fitc (Sigma)-filled NPs (400 μL of a suspension of approximately 1.9 × 10^12^ NPs/mL) were then infused into the carotid artery. The deposition of NPs on mesenteric endothelium was monitored by intravital microscopy for 90 minutes. The amount of NPs bound to the endothelial surface was evaluated by measuring the fluorescence of BSA-Fitc encapsulated in NPs and tNPs. First, four different regions of interest (ROIs) were drawn in the background of the picture, and the mean fluorescence intensity of the background was calculated. This value was subtracted from the starting picture, and then this was converted into a binary picture in order to create a different ROI set on the fluorescent signal. Finally, the Fitc and the background signals were calculated and employed to measure the corrected total cell fluorescence (CTCF):


$$\begin{array}{c} CTCF=\text{Integrated}\;\text{Density}-[\left(\text{Area}\;\text{of}\;\text{Selected}\;\text{Cell}\right)*\\ \left(\text{Mean}\;\text{Fluorescence}\;\text{of}\;\text{Background}\;\text{Readings}\right)]. \end{array}$$

### Prevention by tNP of the binding of IgG from APS patients to β2GPI on rat vascular endothelium

Rats were intraperitoneally injected with LPS (2.5 mg/kg) and then anesthetized with Zoletil (Virbac, 25 mg/kg) and xylazine (Rompun, 7.5 mg/kg). Afterward, they received a slow infusion of Rhodamine 6G (Sigma) into the jugular vein to stain leukocytes and platelets. Targeted or untargeted NPs (400 μL of a suspension of 1.9 × 10^12^ NPs/mL) or saline (400 μL) were then infused into the carotid artery. Ten minutes later, they were then infused into the carotid artery with 6.5 mg of Fitc (Sigma)-labeled IgG purified from five APS patients with medium–high titer antibodies to the Domain I (DI) domain of β2GPI. The deposition of IgG on mesenteric endothelium was monitored by intravital microscopy for 90 minutes. The rate of IgG bound to endothelium was evaluated by comparing the fluorescence intensity on the endothelial surface to the fluorescence intensity of the vascular lumen. First, four ROIs were drawn in the background of the picture, and the mean fluorescence intensity of the background was calculated. This value was subtracted from the starting picture, and then this was converted into a binary picture in order to create a different ROI set on the fluorescent signal. After that, four ROIs were drawn on the blood vessel lumen, and as performed before, this signal was subtracted from the original picture in order to isolate only the most fluorescent spots representing the deposited IgG. Finally, the Fitc and the background signals were calculated and employed to measure the spots-CTCF.

### Prevention by tNP of thrombus formation and vascular occlusion made by APS sera in rat vascular endothelium

The APS model (7) was established in rats that were intraperitoneally injected with LPS (2.5 mg/kg) and then anesthetized with Zoletil (Virbac, 25 mg/kg) and xylazine (Rompun, 7.5 mg/kg). Afterward, they received a slow infusion of Rhodamine 6G (Sigma) into the jugular vein to stain leukocytes and platelets; tNPs, NPs (400 μL of a solution of 1.9 × 10^12^ NPs/mL), and saline were then infused into the carotid artery. Ten minutes later, 1 mL of pooled sera from five APS patients containing antibodies to β2GPI (18) was infused into the carotid artery to cause thrombus formation. The evolution of hemodynamics in the mesenteric vessels was monitored by intravital microscopy for 90 minutes. The number of thrombi observed in the vascular tree and the percentage of occluded vessels were evaluated by measuring the blood flow. A fourth group of rats received 400 μL of a solution of 1.9 × 10^12^ tNPs/mL intravenously 1 day before the above-described procedure, and the same parameters were evaluated.

### Statistical analysis

Statistical significance was determined using the Prism (GraphPad) software. Statistical analysis of means was performed using the two-way ANOVA for the *in vivo* experiments regarding the ability of tNPs to bind activated endothelyum and to block thrombus formation or one-way ANOVA for the *in vitro* tests of NPs and TNPs safety and for the *in vivo* experiment about the ability of tNPs to block APS' IgG endothelial deposition. Variance was similar between groups, and p-values <0.05 were considered statistically significant.

## Results

### Production and characterization of anti-β2GPI-targeted NPs

**Figure 1:**
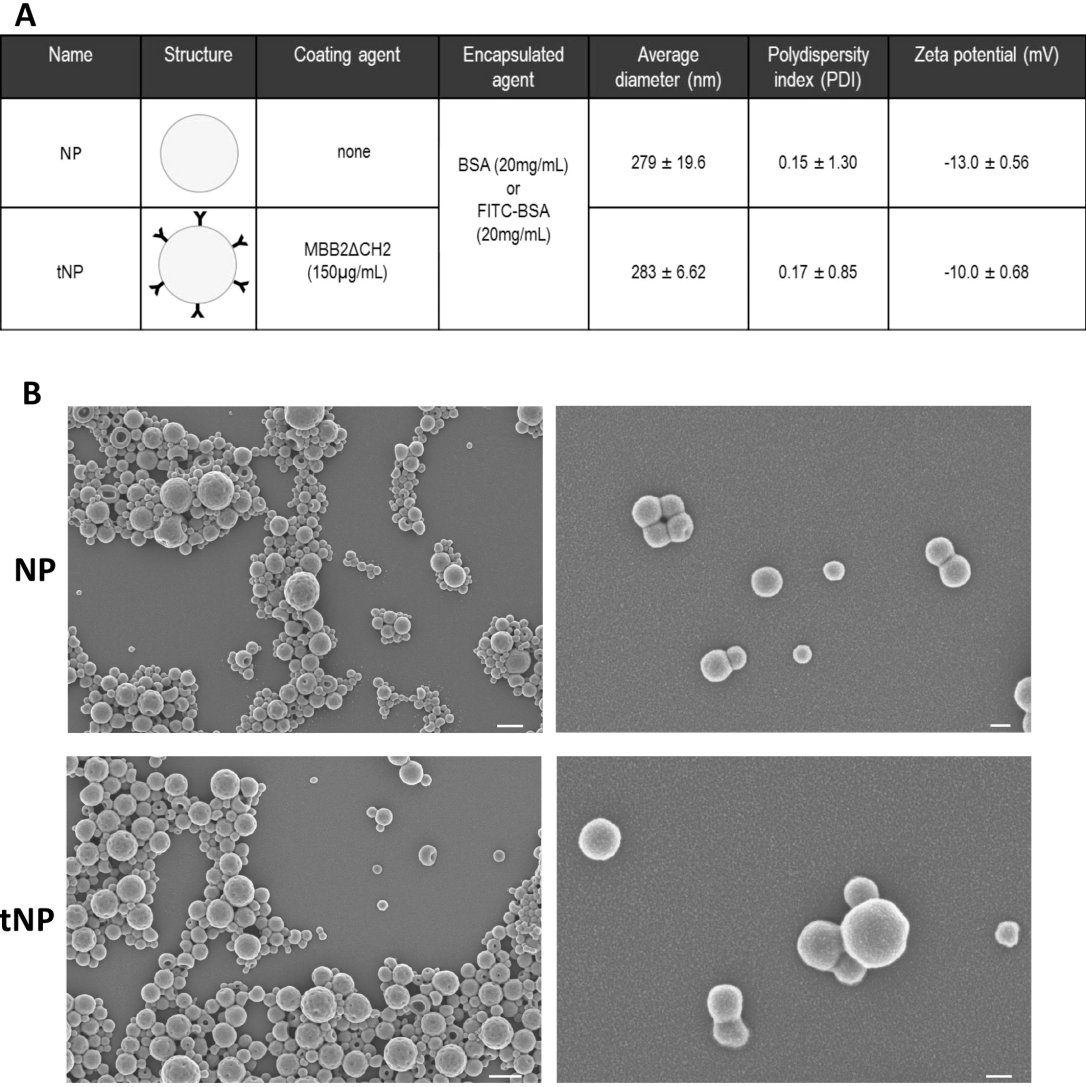
Physicochemical characteristics of untargeted and targeted nanoparticles. **(A)** Untargeted (NP) and targeted nanoparticles (tNP) analyzed by dynamic light scattering (DLS) share similar values of average diameter, polydispersity index (PDI), and particle charge (zeta potential). **(B)** SEM images (scale bars: 500 nm on the left and 100 nm on the right images) show similar morphology of NP and tNP. MBB2ΔCH2 denotes the recombinant CH2-deleted scFv-Fc miniantibody against the DI domain of β2-GPI on tNP.

PLGA–PVA uncoated and targeted nanoparticles (indicated as NPs and tNPs, respectively) were produced using a standardized approach that guarantees a final concentration of approximately 1.9 × 10^12^ NPs/mL, as measured by NTA. Both preparations consisted of an aqueous inner core and a polymeric outer shell, covalently decorated with MBB2ΔCH2, targeting β2GPI, in tNP (20). The core was loaded with Fitc-BSA, to allow visualization of NPs bound to blood vessels by fluorescence microscopy, or BSA, to have comparable nanostructures in experiments aimed at evaluating their therapeutic effect. The physicochemical characterization of NPs is reported in **Figure 1A**. All preparations exhibited a similar size with an average diameter lower than 300 nm (279 ± 19.6 nm for NP and 283 ± 6.63 nm for tNP), a negative surface charge (−13 mV for NP and −10 mV for tNP), and a good index of heterogeneity as confirmed by the low PDI (0.15 for NP and 0.17 for tNP). The regular round shape of the NPs was verified by SEM (**Figure 1B**), showing that MBB2ΔCH2 coupling does not significantly alter NP diameter and morphology. All formulations exhibited very good stability when stored at 4°C for 6 months; no variation in size and zeta potential and no NP aggregation were detected when analyzing preparations by dynamic light scattering (DLS) (data not shown).

### Evaluation of NPs’ safety

**Figure 2:**
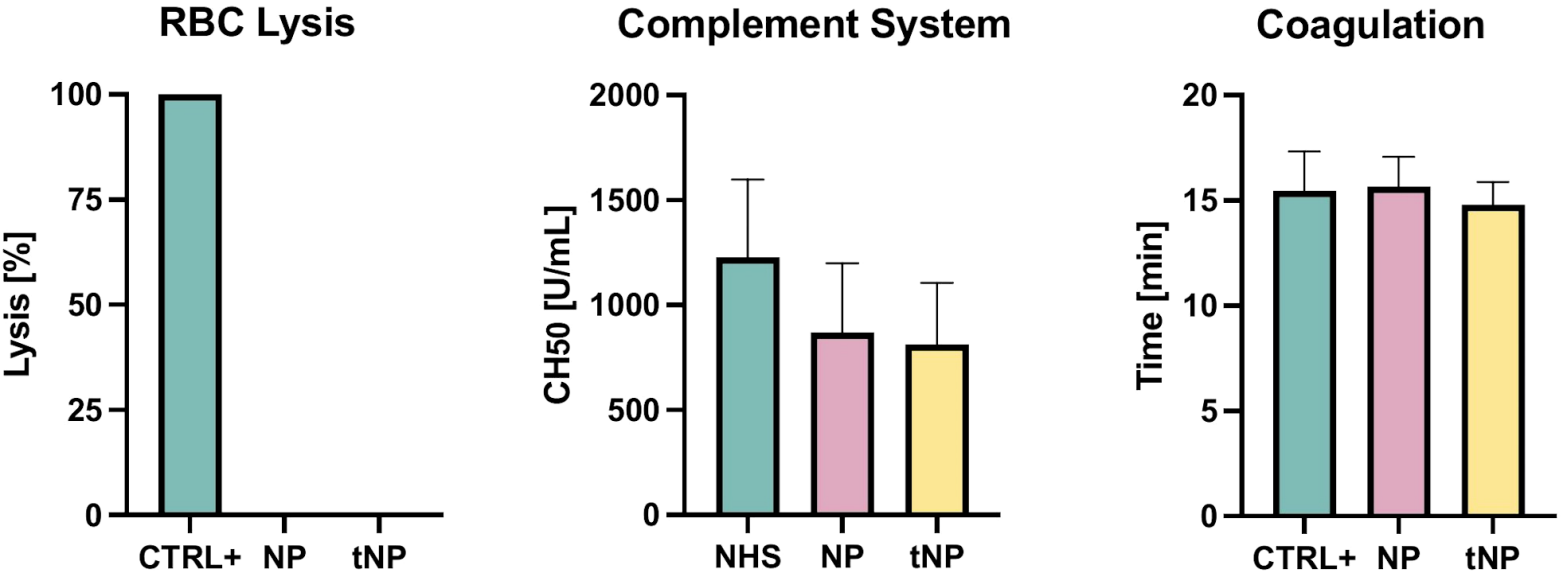
Evaluation of NP safety. RBC Lysis NPs and tNPs did not cause mechanical disruption of red blood cells after 2 hours at 37°C incubation. Complement System NPs were incubated with pooled NHS for 2 hours at 37°C; residual activity of the classical pathway was measured through a hemolytic assay (CH50), showing no significant direct activation of the complement system. Coagulation A turbidity assay, consisting of giving Ca^2+^ to pooled NHP and analyzing turbidity over time, was performed to clarify whether NPs interfere with clotting formation; no significant difference in the half-coagulation time of both NPs and control was documented. The results are presented as mean ± SD. Ns = p-value ≥0.05; one-way ANOVA test.

PLGA–PVA NPs were tested *in vitro* to evaluate their potential safety in circulating blood (**Figure 2**). The possibility that NPs may cause mechanical disruption of red blood cells was investigated by incubating an erythrocyte suspension with NP or tNP for 2 hours at 37°C. No lysis was observed in the supernatant collected at the end of the experiment, thus excluding a direct damaging effect of NPs on red cells. The possible effect of NPs and tNPs on complement activation was then evaluated. Briefly, pooled normal human serum was incubated with each NP at a concentration similar to that used in *in vivo* experiments, and the residual complement activity was analyzed by a hemolytic assay (CH50). The results showed a modest, non-significant reduction of the complement activity, and no difference was found in the effect of the two NPs. A turbidity assay was finally performed to ascertain whether NPs interfere with blood clot formation. To this end, NPs were incubated with pooled NHP, and the sample turbidity was analyzed over time following the addition of a solution containing Ca^2+^ to trigger the coagulation process. The data revealed a similar trend either in the presence or in the absence of NPs, and no significant difference was found between NPs and control groups in the half coagulation time extrapolated from the curves.

### Anti-β2GPI-targeted NPs bind to activated rat mesenteric vascular endothelium

Preliminary experiments were conducted to verify the *in vivo* ability of anti-β2GPI antibodies (recombinant or present in APS patients) to bind to activated endothelium. Serum samples were obtained from five APS patients selected for the presence of medium–high titer IgG to cardiolipin, β2GPI, and, in particular, domain I of β2GPI (**Table 1**); the sera were pooled before use.

**Figure 3:**
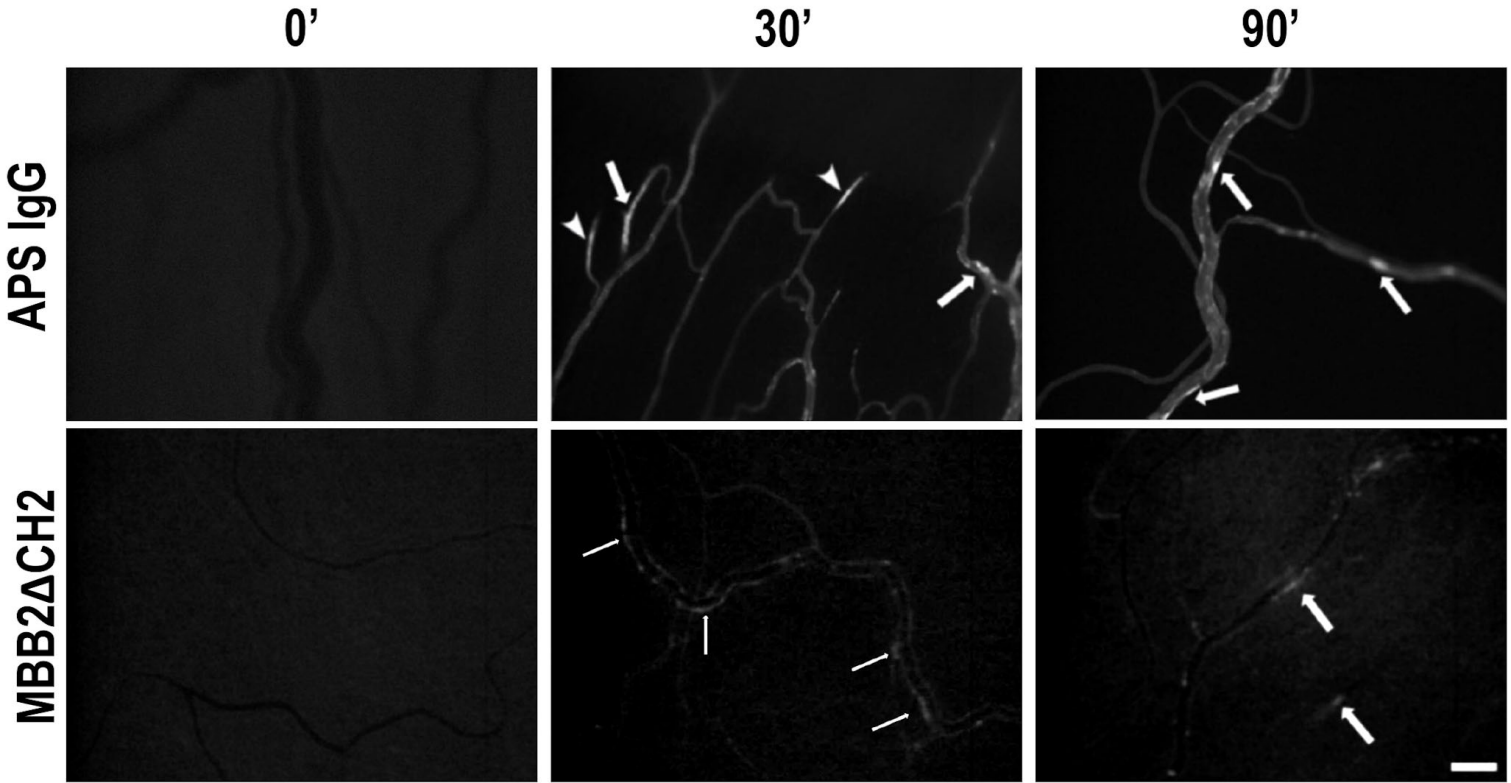
Deposition of anti-phospholipid syndrome (APS) IgG or MBB2ΔCH2 on mesenteric vessels of rats treated with lipopolysaccharide (LPS). The animals were treated with LPS followed by the injection of Fitc-labeled IgG from APS patients or Fitc-labeled MBB2ΔCH2. Antibody deposition in mesenteric vessels was constantly monitored by intravital microscopy for 90 minutes (representative images were obtained at 0, 30, and 90 minutes after antibody infusion). Arrows indicate antibodies bound to the endothelium, while arrowheads indicate formed thrombi. Original magnification ×100. Scale bar 50 μm.

The rats received an intraperitoneal injection of LPS in order to induce binding of β2GPI to endothelial cells, as previously described (21). Fitc-labeled MBB2ΔCH2 or Fitc-labeled APS IgG were administered into the carotid artery, and their localization on mesenteric vessels was monitored over time. Intravital fluorescence microscopy analysis showed a patchy distribution of the fluorescent signal on the mesenteric endothelium of rats receiving either fluorescent MBB2ΔCH2 or aPL IgG (**Figure 3**). As expected, aPL IgG caused vascular occlusions (**Figure 3**), while MBB2ΔCH2, which does not activate the complement system, was able to target β2GPI on endothelial cells but failed to trigger the coagulation process and the vessel occlusion.

**Figure 4:**
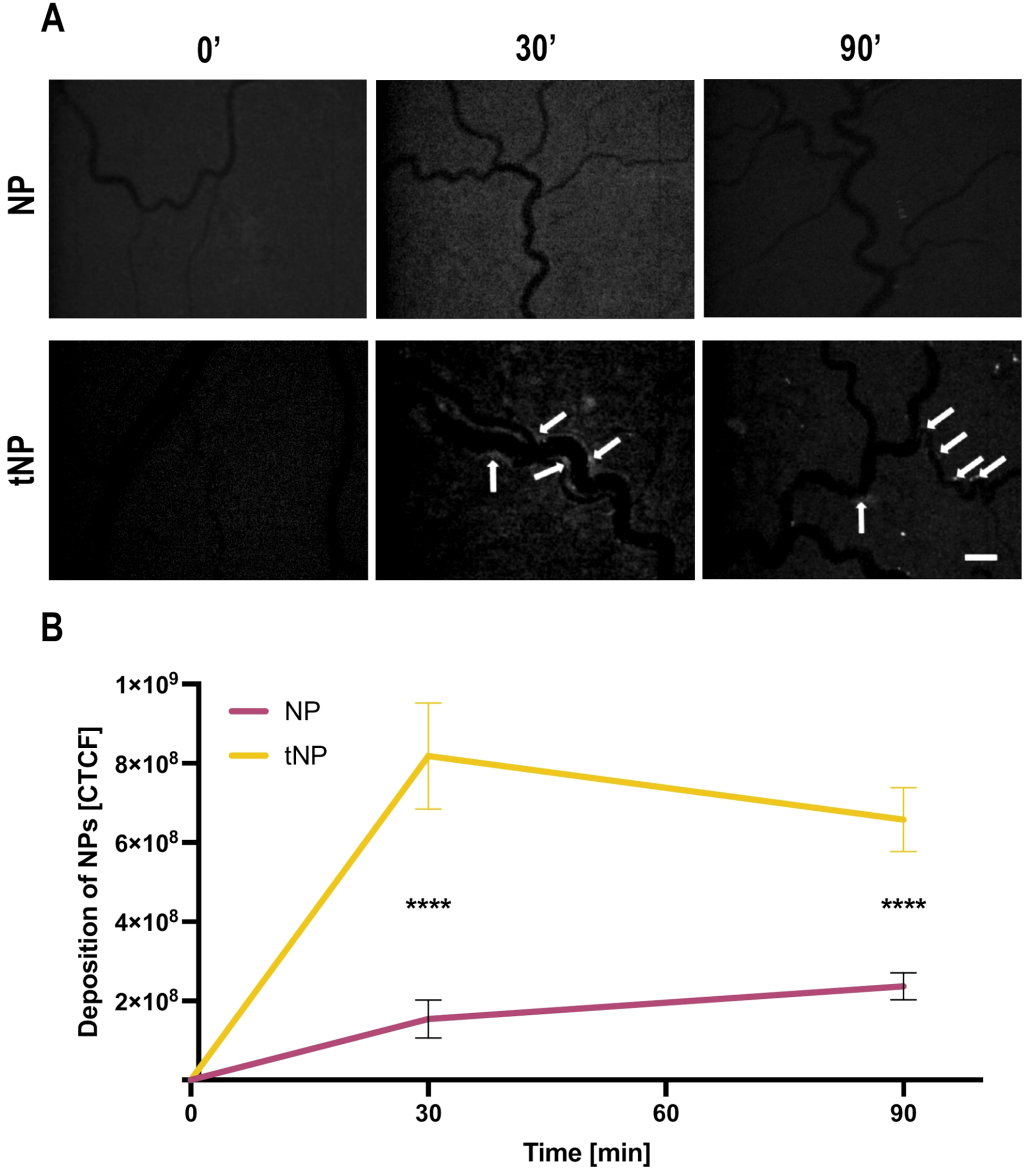
Anti-b2 GPI-targeted NPs are able to bind activated rat mesenteric vascular endothelium. The animals were treated with lipopolysaccharide (LPS) followed by the injection of fluorescent untargeted (NPs) or fluorescent targeted nanoparticles (tNPs). NP and tNP deposition in mesenteric vessels were constantly monitored by intravital microscopy for 90 minutes. **(A)** Representative images obtained at 0, 30, and 90 minutes after nanoparticle infusion. tNPs bound to the endothelium are indicated with arrows. Note the absence of untargeted NBs in mesenteric vasculature. Original magnification ×100. Scale bar 50 μm. **(B)** Quantification of NPs bound to endothelial surface evaluated by measuring the fluorescence of BSA-Fitc encapsulated in NPs and tNPs. A significant deposition of tNPs but not of NPs was measured. The results are presented as mean ± SD. ****p-value ≤0.0001; two-way ANOVA test.

A similar distribution pattern of BSA-Fitc loaded tNPs on the mesenteric endothelial cell was observed in rats treated with LPS, which stimulated cell deposition of β2GPI, recognized by the recombinant anti-β2GPI antibody on NP surface. On the contrary, no relevant fluorescent signal was seen on the mesenteric vessels of rats receiving fluorescent untargeted NPs (**Figure 4A**). The deposition of both types of nanoparticles on vascular endothelium was analyzed by measuring the fluorescence signal of BSA-Fitc encapsulated in the NPs. The results presented in **Figure 4B** show a progressive increase in the deposition of tNPs on the endothelium of blood vessels that started within a few minutes from NP injection, reaching the highest level in 30 minutes and slightly decreased up to 90 minutes. A marginal localization of untargeted NPs was observed on vascular endothelium, and the extent of their deposition was significantly lower than that of tNPs.

It is important to underline that neither NPs nor tNPs were able to activate the coagulation cascade in blood, and intravascular thrombi were not documented during the analysis.

### Anti-β2GPI-targeted NPs prevent deposition of aPL IgG in the APS rat model

**Figure 5:**
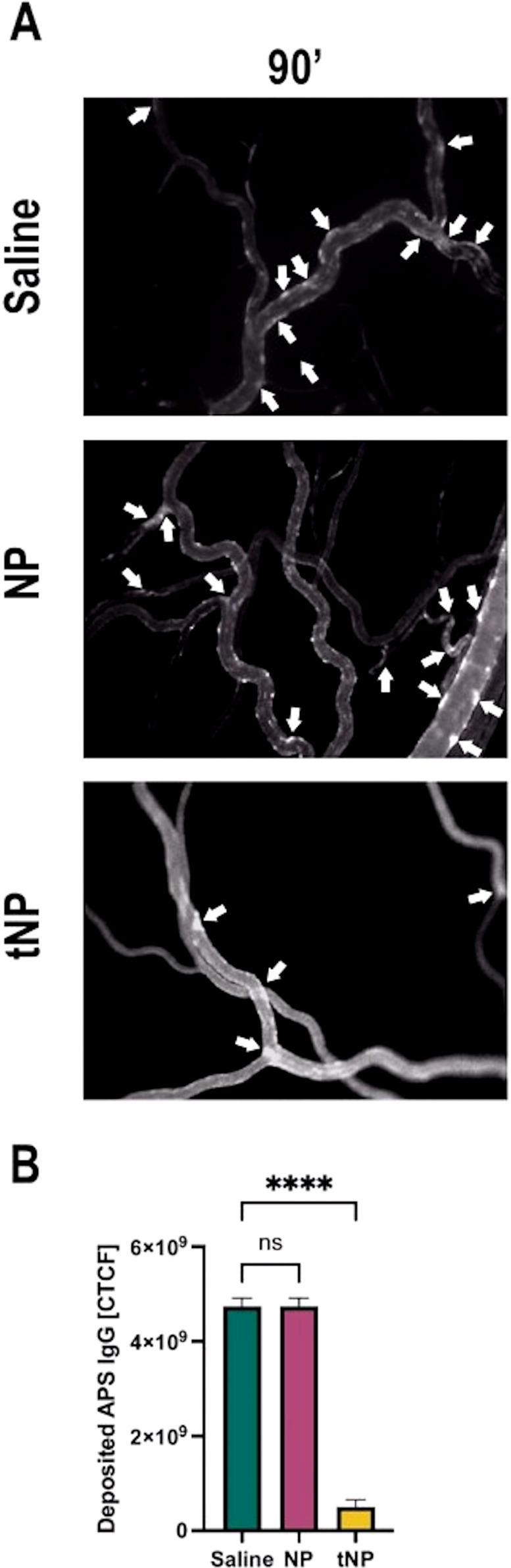
Anti-β2-GPI-targeted NPs prevent anti-phospholipid syndrome (APS) IgG deposits in APS rat model. The animals, after treatment with lipopolysaccharide, received the injection of saline solution, untargeted (NP) or targeted nanoparticles (tNP), followed by the injection of Fitc-labeled IgG purified from APS patients. IgG deposits (indicated with arrows) were constantly observed by intravital microscopy. **(A)** Representative images were collected 90 minutes after the injection of labeled IgG. **(B)** The amount of IgG bound to the endothelial cell surface was evaluated by measuring the fluorescence of Fitc-labeled IgG. The results in panel B are presented as mean ± SD. ****p-value ≤0.0001; one-way ANOVA test. NS, Not significant.

To examine the effect of tNPs in preventing the binding of aPL to blood vessels, LPS-treated rats received targeted or untargeted NPs, or saline as a control, followed by a pool of Fitc-labeled IgG purified from five APS patients, containing antibodies to β2GPI. The deposition of IgG on mesenteric blood vessels was monitored over time by intravital microscopy (**Figure 5A**). The analysis showed again an irregular distribution of Fitc-labeled IgG on endothelial cells, and the sites along the vessels, where the labeled IgG were deposited, had decreased in number in rats treated with tNPs compared to those seen in rats receiving saline or untargeted NPs (**Figure 5A**). Aware of the fact that the number of deposits alone was not exhaustive in describing the phenomenon observed, a quantitative analysis of fluorescence intensity was performed as indicated above. As shown in **Figure 5B**, the deposits of aPL IgG on endothelial cells were markedly reduced and nearly undetectable 90 minutes after the administration of tNP compared to those seen in animals receiving saline. Interestingly, some inhibition of IgG binding to endothelial cells was observed in rats treated with untargeted NPs, most likely due to non-specific absorption of circulating IgG by the NPs rather than the result of competitive inhibition of IgG binding to endothelial cells.

### Effect of targeted NBs on *in vivo* thrombus formation

**Figure 6:**
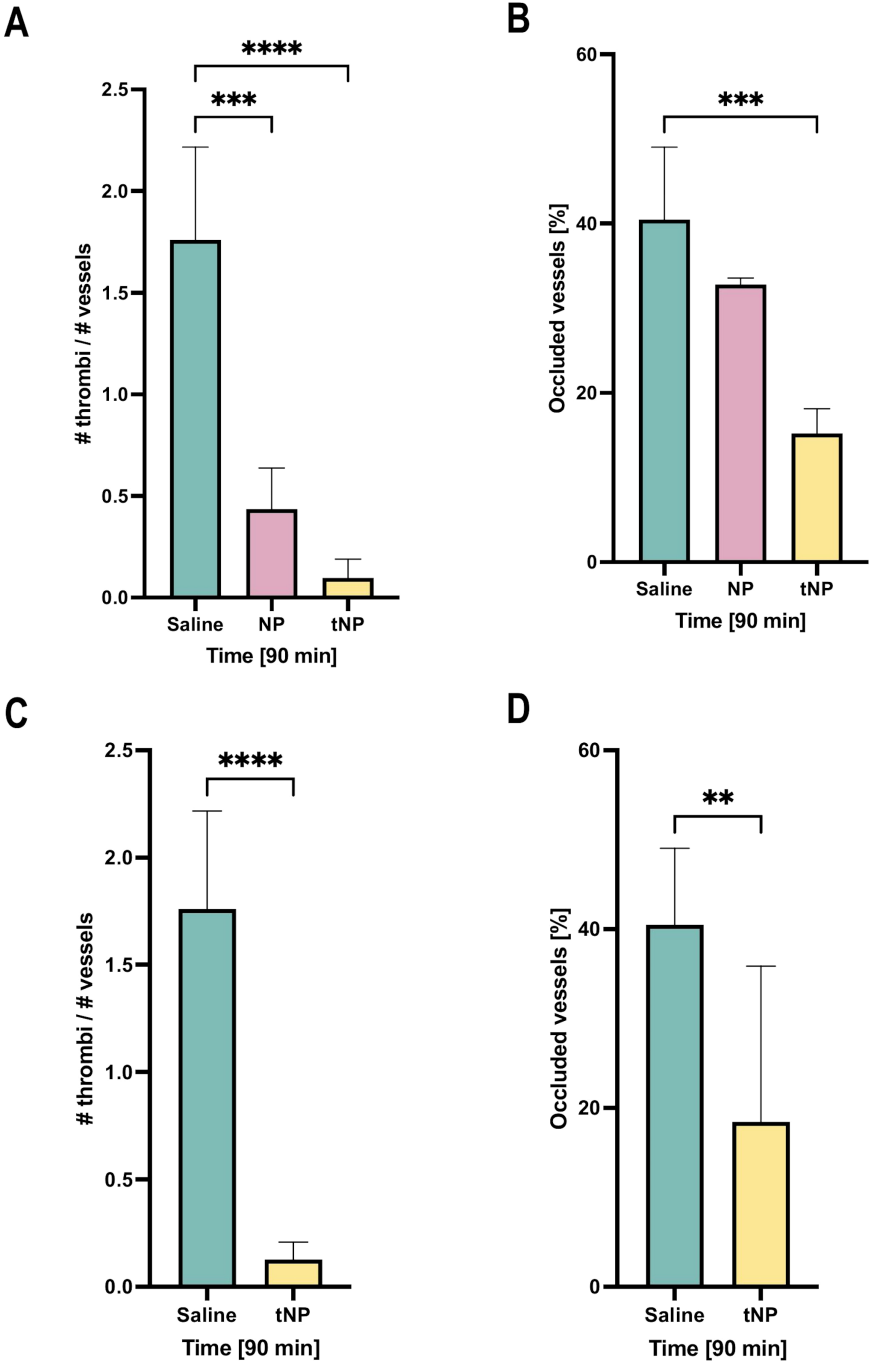
Effect of targeted NBs on thrombus formation in the rat model of anti-phospholipid syndrome (APS). A first group of rats, after treatment with lipopolysaccharide, received the injection of saline solution, and untargeted (NP) and targeted nanoparticles (tNP) followed by the injection of APS sera; thrombus formation and vascular occlusions were observed by intravital microscopy for 90 minutes, showing that NPs markedly reduced the formation **(A)** and the dimension **(B)** of intravascular thrombi compared to saline treatment. A second group of rats received the injection of saline solution and tNPs 24 hours before lipopolysaccharide (LPS) and APS serum treatments; animals were observed by intravital microscopy showing that tNPs still reduced the formation **(C)** and the dimension **(D)** of intravascular thrombi compared to saline treatment, more significantly after 90 minutes after sera injection. The results are presented as mean ± SD. ****p-value ≤0.0001; ***p-value ≤0.001; **p-value ≤0.01; two-way ANOVA test.

The ability of NPs to prevent the thrombotic activity of deposited IgG anti-β2GPI was then examined. To this end, rats primed with LPS, to promote deposition of β2GPI on endothelial cells, received either targeted or untargeted NPs, or saline as a control prior to administration of pooled sera from five APS patients containing antibodies to β2GPI to trigger coagulation. Analysis of the mesenteric vasculature, monitored over time by intravital microscopy, revealed a marked decrease in the number of thrombi in tNP-treated rats compared to animals receiving saline (**Figure 6A**). A reduced number of blood clots were also seen following administration of untargeted NPs, consistent with the results of passive absorption of circulating aPL IgG shown in **Figure 5B**, although the decrease was less marked than that observed in tNP-treated rats (**Figure 6A**). Interestingly, the analysis of the blood clot dimension, which may be responsible for vascular occlusion, revealed that only tNPs have a significant effect in reducing the number of occluded vessels while untargeted NPs were almost ineffective (**Figure 6B**).

To investigate whether the tNPs, administered several hours prior to patients’ aPL IgG, circulate in sufficient numbers to prevent coagulation, the rats were challenged with tNPs or saline 24 hours before LPS priming followed by administration of aPL IgG. The results presented in **Figures 6C, D** show that tNPs were still able to significantly reduce both the number of blood clots and the percentage of occluded vessels.

## Discussion

Polymeric nanoparticles coated with a monoclonal antibody to β2GPI selectively targeting endothelial cell-bound β2GPI were effective in preventing thrombus formation and vessel occlusion in a rat model of APS. These results extend our previous observation of rapid elimination of vessel-occluding clots obtained with nanobubbles coated with recombinant tissue plasminogen activator and a monoclonal antibody to β2GPI but delivered only after thrombus formation (20).

The nanoplatform proposed in this study is based on PLGA/PVA, biodegradable and biocompatible synthetic polymers, already approved by the Food and Drug Administration (FDA) and European Medicines Agency (EMA) as delivery systems for therapeutic agents (22) and has been subjected to clinical trial (23); today, drugs that use this platform as nano/microparticle structure are commercially available for cancer therapy, to reduce inflammation, to treat pain or neurological diseases, or for vaccination (24). Nanoparticles showed a regular round shape with a diameter of approximately 280 nm surrounded by a polymeric outer shell, as previously documented (17, 25, 26). The aqueous inner core can be loaded with fluorescent dyes (16), for their visualization, or drugs (17, 27–29), for specific delivery to selected targets. The safety of NPs was confirmed by their failure to lyse red blood cells or to interfere with coagulation or complement systems and, *in vivo*, following their injection in rat circulation. In this study, we focused on the results obtained by the injection of 0.4 mL of NP suspension containing approximately 0.76 × 10^12^ NPs. The dose was derived from a previous study in mice (30, 31) and was adjusted taking into consideration the different body weights of the animals.

The *in vivo* biodistribution of Fitc-labeled aPL IgG in rats was observed by intravital microscopy that revealed their deposition on the mesenteric endothelium with a patchy distribution. This fluorescent pattern is consistent with our previous findings (18, 19) and is most likely the result of the uneven deposition of circulating β2GPI on endothelial cells. Studies have shown that the binding of β2GPI to endothelial cells occurs independently of the presence of antibodies and requires priming with LPS, followed by the interaction of cell-bound β2GPI with specific auto-antibodies preformed in the serum, providing another hit for activation of the complement activation and the coagulation cascade (7, 21). A similar priming effect can be induced by pregnancy, characterized by β2GPI deposition on trophoblast and decidual endothelial cells (21, 32). Antibody clustering in areas of β2GPI localization favors complement activation that plays a critical role in triggering activation of the coagulation cascade, as suggested by the failure of aPL IgG to induce thrombus formation in C6-deficient rats and animals treated with a neutralizing antibody to C5 (7).

The distribution pattern of the monoclonal antibody to β2GPI (MBB2ΔCH2) on endothelial cells was similar to that of aPL IgG, further supporting the conclusion that β2GPI deposited on endothelium is a potential target of circulating aPL antibodies in APS patients. MBB2ΔCH2 was selected to coat NP as a targeting agent for cell-bound β2GPI for several reasons. First, this monoclonal antibody recognizes a neoepitope on domain I of bound β2GPI, regarded as the main target of pathogenic aPL antibodies responsible for thrombosis and fetal loss in animal models of APS (7, 14, 18, 33). MBB2ΔCH2 binds to β2GPI equally well as MBB2 but, unlike the parent molecule, is unable to activate the complement system due to the deletion of the C1q fixing CH2 domain of the antibody and therefore fails to trigger the coagulation cascade (14). Finally, the recombinant antibody has a higher affinity than patients’ antibodies for the target antigen and can compete with them for their interaction with cell-bound β2GPI (14).

NPs coated with MBB2ΔCH2, targeting β2GPI, bound to endothelial cells within 30 minutes from injection and were effective in preventing *in vivo* binding of IgG purified from APS patients to endothelial cells and their prothrombotic activity. These results were obtained using an amount of NP-bound MBB2ΔCH2 20 times lower than that of soluble antibody required to obtain a similar effect. Moreover, the tNPs were persistently present on the endothelium through the experimental procedure and contributed to protection from thrombosis induced by patients’ IgG by competing with them, as a result of the higher affinity of MBB2ΔCH2 and also by steric hindrance, as they cover a larger surface area than the soluble antibodies.

The medical need behind the project is to develop a novel targeted therapy to prevent thrombus formation in APS. The use of tNPs for long-term treatment of APS patients, in the absence of events triggering coagulation, is probably not preferable to the standard anti-coagulant therapy, although the latter treatment is not free from recurrent thrombosis reported in 30%–40% of high-risk patients with triple APL positivity (10). The tNPs may be better indicated as first-line therapy of APS patients in acute situations, such as inflammatory processes, acute infections, and vascular surgery, that promote coagulation by activating the endothelial cells (34). We have previously shown that re-thrombosis was successfully prevented by treating an APS patient undergoing femoral popliteal bypass for thrombotic vascular occlusion with eculizumab to inhibit complement activation (6). MBB2ΔCH2-coated NPs have the advantage of acting upstream of complement inactivation, competing with aPL for binding to endothelial cells and remaining cell-bound throughout the experimental procedure. This approach could also be effective in other surgical situations involving APS patients, like the preparation of arteriovenous fistulas in dialysis patients, cardiac valvular diseases, or diagnostic biopsies.

A preventive approach is typically studied by injecting the drug just before the stimulus, which is not always feasible in a clinical setting, considering also the drawbacks of long-term anti-coagulation (35).

For these reasons, we also tested the preventive effect of injecting tNPs 24 hours before the challenge with antibodies from APS patients; this period represents also the initial elimination of polymeric NPs, mainly dependent on liver macrophages (36), that can induce a fast clearance from the circulation; a single injection of tNPs was still able to prevent thrombus formation and vascular occlusion for all durations of the study.

In conclusion, anti-β2GPI-targeted polymeric nanoparticles represent a stable and safe approach to preventing thrombus formation and vessel occlusion in a rat model of APS caused by patients’ antibodies.

## Data Availability

The raw data supporting the conclusions of this article will be made available by the authors, without undue reservation.
